# Aliphatic Diacidic Long-Chain C16 Polyesters from 10,16-Dihydroxyhexadecanoic Acid Obtained from Tomato Residual Wastes

**DOI:** 10.3390/molecules24081524

**Published:** 2019-04-17

**Authors:** Daniel Arrieta-Baez, José Vicente Hernández Ortíz, José Campos Terán, Eduardo Torres, Mayra Beatriz Gómez-Patiño

**Affiliations:** 1 Instituto Politécnico Nacional—CNMN, Unidad Profesional Adolfo López Mateos, Col. Zacatenco, México City, CDMX CP 07738, Mexico; danielarrieta@hotmail.com; 2 División De Ciencias Naturales y de Ingeniería, Universidad Autónoma Metropolitana Unidad Cuajimalpa, México City, CDMX CP 05300, Mexico; chendf_23@hotmail.com (J.V.H.O.); jcampos@correo.cua.uam.mx (J.C.T.); 3 Centro de Química y Facultad de Ingeniería Química, Benemérita Universidad Autónoma de Puebla, Puebla CP 72570, Mexico; eduardo.torres.ramirez@gmail.com

**Keywords:** polyesters, lipases, MALDI-TOF, tomato, agroresidual waste

## Abstract

10,16-dihydroxyhexadecanoic acid obtained from agroresidual tomato waste, was oxidized to produce 7-oxohexadecanedioic acid in good yield (>70%) and purified without oxidation side products in one step. Polycondensation with 1,8-octanediol, yielded the polyester (poly(*ω*-carboxyl PA-*co*-OD)) with M_w_ = 2155.15 and M_n_ = 1637.27. The best enzymatic reaction conditions to get the polyester were using lipase CAL-B (%-by-wt relative to monomer) in toluene as a solvent for 1 h at 60 °C. The poly(*ω*-carboxyl PA-*co*-OD) was characterized by ^1^H- and ^13^C-NMR, mass spectrometry (MALDI-TOF) and the polyester film formed with a Langmuir-Blodgett Trough was analyzed by means of spectroscopic ellipsometry and atomic force microscopy.

## 1. Introduction

Plastic pollution concerns caused by the use of petroleum-based plastics have led to increased attention to aliphatic polyesters as a possible solution, due to their biodegradable characteristics, as well as their specialized use in the biomedical field as drug delivery vehicles and tissue scaffolds [1,2,3]. In fact, aliphatic polyesters are the most promising families of renewable polyesters to be used in medical fields because of their unique performance, satisfactory biocompatibility and good biodegradability. Besides, due to their flexible physical and chemical properties, they are considered the most competitive biodegradable polymers commercialized up to now used for a wide range of applications in day to day life [4,5,6,7,8]. The physical properties of these copolyesters and their degradability are significantly influenced by the incorporation of different diols and diacids in the main chain and also depend on the crystallinity, the chain length and hydrophilicity/hydrophobicity balance [9]. The presence of functionalized groups such as hydroxyl and carboxylate in polyesters can increase their hydrophilicity and degradability due to the susceptibility of the ester bond to hydrolysis. Moreover, these groups could provide an anchor point for a variety of bioactive molecules such as drugs, peptides, and nutraceuticals. Using functionalized monomers in the polycondensation reaction can lead to new biomaterials with a significant role in biological activities [10,11,12].

Effective examples using diols and dicarboxylic acids polyester have been described as a drug delivery vehicles and tissue scaffolds in the medical field [13,14], as plasticizers [15] and some of them are used as food packing materials nowadays [16]. Most of these polyesters have been prepared using short-chain monomers and very few works have focused on polyesters with 16 carbon atoms or above, even though they have been demonstrated to be non-cytotoxic in cell culture and biocompatible upon implantation in animal models [17]. Cutin and suberin are important plant barrier biopolymers and they are formed mainly of long-chain fatty acids. Cutin is the main polyester present in the skin of fruits, leaves, seeds, and green stems of higher plants. Mixtures of C_16_ and C_18_ polyhydroxy acids have been isolated and 9(10),16-dihydroxyhexadecanoic acid, is characterized as the most abundant [18,19,20]. The monomeric composition and interesting chemical structure of the monomer makes of this natural polyester a raw material to obtain “green” chemicals to produce bioplastic for specific uses.

We have successfully isolated the main monomer of tomato cutin, 10,16-dihydroxy-hexadecanoic acid (10,16-DHPA), from agro-industrial residual wastes in good yield (≈ 40% from cutin), and used it in polyesterification and trans-esterification lipase-mediated reactions [21]. 10,16-DHPA can be oxidized to produce a C_16_ dicarboxylic acid [20]. Natural long-chain dicarboxylic acids could be an interesting and useful group of monomers to design and synthesize unique functional polyesters that would be also biodegradable [9]. However, it is really difficult to synthesize those present in cuticles (mainly 9,10-epoxyoctadecanedioic, 1,18-*cis*-9-octadecenedioic, and 9,10-dihydroxyoctadecanedioic acids) by chemical methods, and there are only a few examples using microorganisms [6,22].

In the present work, we used a very soft oxidation reaction to obtain 7-oxohexadecanedioic acid (ω-carboxyl oxo PA) in good yield. The synthesized diacid was then polycondensed with 1,8-octanediol to get a polyester with a weight-average molecular weight (M_w_) of 2155.15 and a number-average (M_n_) of 1637.27. A polyester film was prepared with a Langmuir-Blodgett Trough and analyzed with spectroscopic ellipsometry and AFM. The obtained bio-copolyester showed interesting characteristics that make it a potential biomaterial to be used in the biomedical field, especially as a drug or nutraceutical delivery vehicle.

## 2. Results and Discussion

Since biopolymers are biodegradable and the main products are obtained from renewable resources such as agro-resources, they represent an interesting alternative route to common non-degradable polymers. In this regard, biomass represents an abundant carbon-neutral renewable resource for the production of biomaterials. The tomato fruit agro-residues represent approximately 20% of losses in crops and management in supply centers and could be used to obtain the 10,16-dihydroxyhexadecanoic acid (10,16-DPHA, **1**), the main monomer of tomato cutin. 10,16-DHPA (**1**) can be modified to obtain different monomers that can be used to produce different unique polymers [20].

### 2.1. Oxidation of 10,16-DHPA (**1**).

**Scheme 1:**
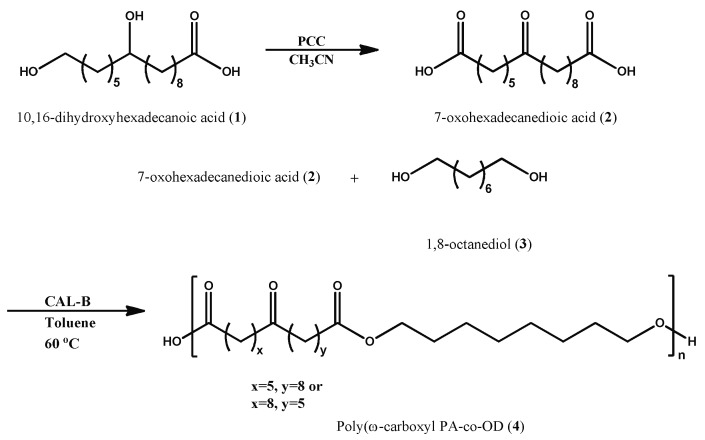
Lipase-catalyzed polycondensation of 10,16-DHPA and 1,8-OD.

10,16-DHPA was obtained and characterized according to our previous work [20]. Then, it was oxidized using PCC to produce the corresponding long chain dicarboxylic acid in good yield (73%; Scheme 1). The NMR and MS data were compared with those previously reported and the product was confirmed as 7-oxohexadecanedioic acid (**2**) [20].

Long-chain aliphatic α,ω-dicarboxylic acids (diacids) are an important group of chemicals used in the production of different plastics, and other chemical applications as well. However, most of these compounds are obtained by different complex chemical reactions that involve the use of non-renewable feedstocks, multi-step reactions processes that generate harmful byproducts and afford low yields of diacids. Under these characteristics, the production of dodecanedioic acid has been done using butadiene, a non-renewable petrochemical feedstock, as starting material. Diacids obtained by this process are limited to four-carbon multiple lengths. Dodecanedioic acid is the only long-chain aliphatic diacid obtained from an industrial process. Other diacids greater than 13 carbons are difficult to prepare on a large scale, and possible processes are being investigated. Therefore, the use of ω-hydroxy acids, such as the 10,16-DHPA, represent a renewable source to get long chain functionalized diacids using a mild oxidation process.

### 2.2. Enzymatic Polycondensation

In order to study the effect of the diol chain in the reaction, different compounds were assayed. However, only 1,8-octanediol gave a good yield, while with other diols the polycondensation reaction was very poor and in some cases, there was no reaction observed (data not shows). Therefore, 1,8-octanediol was used to study the polyester reaction. First, the effect of the reaction temperature on the yields and molecular weights in the Novozyme 435-catalyzed polycondensation was studied. To detect the temperature dependence of this reaction, toluene was used as a model substance. It is well known that Novozyme 435 is a very thermostable enzyme preparation. Thus, the reactions were carried out at 60, 70 and 80 °C. The yields of the reactions (%) and the molecular masses (*m*/*z*) of the polyesters obtained are shown in Table 1, and the best results were achieved at 60 °C. Accordingly, all the following reactions were done at 60 °C. Longer reaction times led to no distinct increase of the molecular masses. Thus, all further polycondensations were stopped after 24 h. Molecular weights and polydispersities were determined by MALDI-TOF analysis.

**Figure 1:**
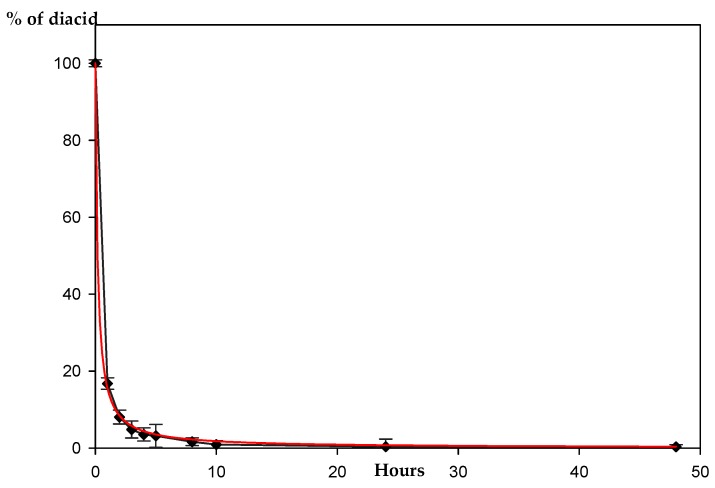
ω-Carboxyl oxo PA consumption in the enzymatic copolymerization reaction with 1,8-OD.

Some studies of the polycondensation reactions between aliphatic long-chain diacids and diols are carried out with Novozyme 435 10%-by-wt relative to monomer, and higher molecular masses are achieved at longer times (48–72 h) [23]. However, we observed that during this time there were very small changes in the molecular weight and the polydispersity index, so we decided to analyze the lipase-polyesterification reaction at shorter times during the first 48 h. Figure 1 shows that CAL-B is highly active for the copolymerization between ω-carboxyl oxo PA and 1,8-octanediol, and it is very clear that ω-carboxyl oxo PA is completely consumed in the first 10 h. However, between 20 to 72 h reaction times, there were very small changes in the reaction.

Yang et al. [12], and Azim et al. [23], have reported that monomer consumption is higher in the first 10–12 h. On the other hand, Feder and Gross [24], mention that CAL-B catalyzed-reactions without molecular sieve or vacuum, the increment in the chain length is very fast and the consumption of the monomers is very high.

**Figure 2:**
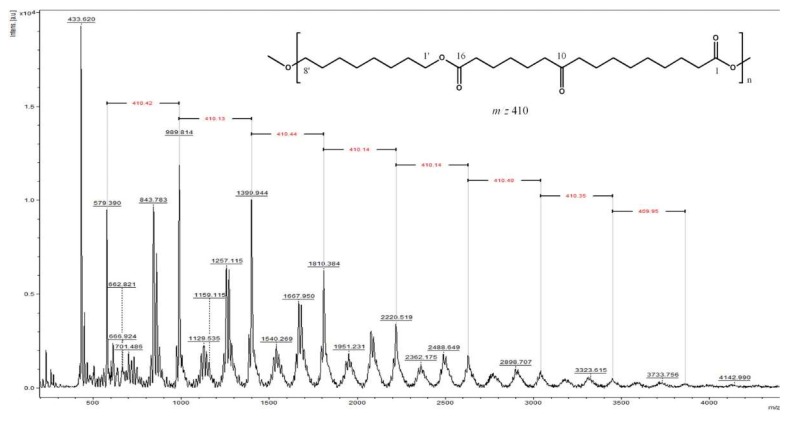
MALDI-TOF spectra of the polycondensation reactions.

The time course of changes in the weight-average molecular weight (M_w_) and polydispersity index (M_w_/M_n_) for N435-catalyzed polycondensations of 1,8-octanediol with ω-carboxyl oxo PA to prepare poly(ω-carboxyl PA-co-OD) are displayed in Table 2. Higher poly(ω-carboxyl PA-co-OD) M_w_ was achieved in the first hour. Beyond 1 h, further increases in molecular weight occurred slowly. The MALDI-TOF spectrum is shown in Figure 2.

According to the MALDI-TOF spectrum, an *m*/*z* 410 peak was assigned as a repetitive unit which belongs to 1,8-octanediol with a ω-carboxyl oxo-PA. At least eight repetitive units were detected in the spectrum for an M_w_ 2155.15 and M_n_ 1627.37 at 1 h (Figure 2), using a DHB (dihydroxybenzoic acid) matrix.

### 2.3. NMR Characterization

**Figure 3:**
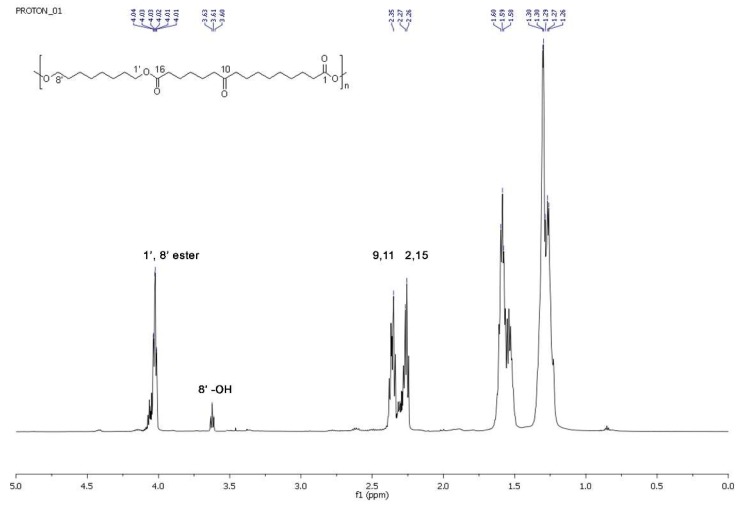
^1^H-NMR spectra of the polycondensation reactions.

To ensure that conversion of *ω*-carboxyl-oxo PA and 1,8-octanediol to polyester occurred completely, ^1^H- and ^13^C-NMR spectra of the corresponding poly(*ω*-carboxyl PA-*co*-OD) were recorded. Chemical shifts of the polyester are listed in the Materials and Methods section. The ^1^H-NMR spectrum of the polyester showed a triplet at δ 4.02 ppm, indicating the presence of an ester bond (-CH_2_-O-CO-; Figure 3). This was confirmed by the correlation in the HMBC of these protons with the carbonyl group at δ 173.9 ppm.

### 2.4. Langmuir-Blodgett Monolayer Formation

The structural analysis of the obtained co-polymer demonstrates a linear structure that makes it a model compound able to carry out the formation of a monolayer. There is a great interest in the construction of systems formed by molecules that interact with each other as parts of a machine. These are the so-called organized molecular assemblies, where the monolayer is produced in the air-water interface by spreading the molecules in an appropriate way, as described in the literature, to be subsequently fixed on solid support forming supramolecular structures with defined characteristics.

**Figure 4:**
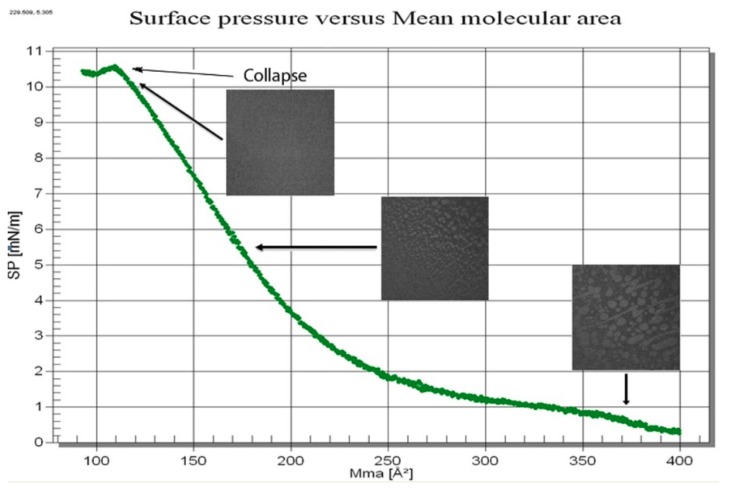
Surface pressure (mN/m)-area (Å2) isotherm of the copolymer at 24 °C on a pure-water subphase.

Surface pressure-area isotherm measurements are the conventional way to characterize the phase behavior of Langmuir-Blodgett monolayers. Figure 4 shows the surface pressure-area isotherm of poly(*ω*-carboxyl PA-*co*-OD) at 24 °C on a pure water subphase. A number of distinct regions are shown in this isotherm. This isotherm shows a phase transition to a low surface pressure (Π < 1 mN m^−1^) to reach a value of approximately 250 Å^2^ per molecule. As the monolayers are being compressed, there is an increase in surface pressure until reaching a maximum value of 10.5 mN m^−1^, with a specific molecular area average of 110 ± 18 Å^2^ per molecule.

As we can see in Figure 4, Brewster Angle Microscopy (BAM) images show irregular areas with bright and dark zones. When the surface pressure increase from 0.48 to 5.5 mN m^−1^ the brightness zone start breaking to the smallest and much better-organized domain. This effect could be related to an aggregation process in the air-water interphase under compression. Finally, at 10.3 mN/m the film is more homogeneous without aggregates and dark zones indicating a reducing porosity. The monolayer of the copolymer obtained under these conditions was transferred to mica for further studies by means of spectroscopic ellipsometry and AFM.

### 2.5. Atomic Force Microscopy and Spectroscopic Ellipsometry Analysis

**Figure 5:**
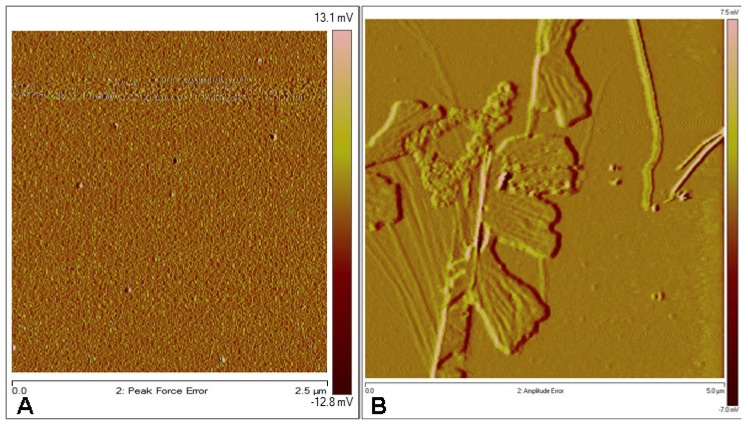
Atomic force microscopy image of the Langmuir-Blodgett monolayer (**A**) and part of the monolayer in the border of the film (**B**).

Subsequently, an atomic force microscopy (AFM) analysis was carried out. From the analysis by AFM, we can observe the formation of a film with a good organization and a high degree of order that gives rise to a homogenous and compact monolayer (Figure 5a). Even when the film is very homogenous, small porous or cavities are perceived to be distributed along the surface. This characteristic could be attributed to some grade of disorder on the linear long-chains. It was difficult to perceive the Langmuir-Blodgett monolayer and the mica, so we searched a part of the monolayer that would be uneven (Figure 5b), to differentiate between the monolayer and the substrate (mica) in order to determine the thickness of the monolayer through this technique. An average thickness of 9.1 nm was determined from an analyzed area of 5 × 5 μm^2^.

**Figure 6:**
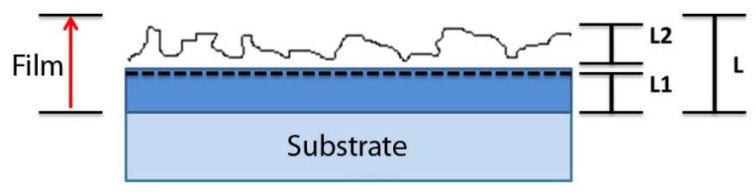
Model of the copolymer film thorough spectroscopic ellipsometry analysis (L1: dense layer, L2: rough layer).

From the studies performed by spectroscopic ellipsometry, it was determined that the Langmuir-Blodgett monolayer has a thickness of 8.794 nm, observing a thickness of the dense layer of 8.135 nm and 0.659 nm corresponding to the rough layer of the film (roughness of the copolymer of 72% in the rough layer) (Figure 6).

It should be noted that the analysis performed by spectroscopic ellipsometry is more accurate than that of AFM, because in this last technique the analysis of the topography was performed in the periphery of the monolayer, where we found the irregularity that leads to a small error in the exact determination of the thickness of the film, while in the analysis by spectroscopic ellipsometry the determination of thickness was made in the center of the film, where it is homogeneous.

## 3. Materials and Methods

### 3.1. Materials

Toluene and 1,8-octanediol were purchased from Sigma–Aldrich (Toluca, Mexico) and used without further purification. Novozymes 435, which is immobilized on a macroporous acrylic resin (abbreviated as CAL-B, specified activity 10,000 PLU/g) was purchased from Novozymes (São Paulo, Brasil). All other chemicals not listed above were of analytical grade from Sigma-Aldrich.

### 3.2. Methods

Copolyesters obtained from the lipase-catalyzed reactions were characterized by ^1^H- and ^13^C-NMR (750 MHz Ascent NMR System; Bruker, Billerica, MA, USA). The NMR spectra were recorded in deuterated chloroform (CDCl_3_). To determine molecular mass and molecular mass distributions, a Matrix-Assisted Laser Desorption/Ionization Time-of-Flight Mass (MALDI-TOF) analysis was carried out using a Bruker Autoflex Spectrometer. A solution of approximately 1 mg/mL of copolymers in chloroform was used with different matrices in a volume ratio of 4:1 to 5:1. MALDI-TOF conditions were varied widely to obtain optimal spectra by adjusting laser power (30−90%), reflectron voltage gain (12–60x, and pulsed ion extraction (10–300 ns). The best results were obtained using dihydroxybenzoic acid (DHB) organic salt as a matrix. Polymeric distributions with the repeating unit of 410 Da were found in the region 500 to 3500 *m*/*z* for the soluble polymers. Electrospray Ionization (ESI) analysis was done on a Bruker micrOTOF-Q II (Bruker Daltonics, Billerica, MA, USA). Samples were dissolved in methanol and were injected directly to the spectrometer. The polymer related peaks were found in positive and negative ion mode (ESI+ or ESI−). The capillary potential was −4.5 kV, the dry gas temperature 200 °C and the drying gas flow 4 L/min. Total ion chromatograms from *m*/*z* 500 to 3000 were obtained. MS data were processed using PolyTools v1.0 (Bruker Daltonics).

#### 3.2.1. Langmuir-Blodgett trough

A KSV minitrough (KSV Instruments, Helsinki, Finland) was used to obtain the surface pressure-area isotherm. A rectangular trough of 98 cm^2^ made of Teflon and a subphase of 57 mL of circulating pure water in channels laced underneath the trough at a regulated temperature of 24 (± 0.5 °C). In order to provide symmetric film compression, two mobile Teflon barriers were employed and the surface pressure was continuously monitored with a platinum bar sensor as a tensiometer (0.01–1 mN/m of resolution) located on an anti-vibration table (Accurion Halcyconics-i4 M6/25; Accurion, Goettingen, Germany) and in a dust-free environment. A stock chloroform solution of a diacid-diol copolymer with a concentration of 1 mM was used. The solution was kept in a freezer at −20 °C when not being used. Using a Hamilton syringe, the copolyester solution was deposited dropwise on the subphase surface and after 10 min of solvent evaporation a constant compression was started. Compression speed used was 10 mm/min, corresponding to a 5 mm/min barrier moving speed. When the copolymer monolayer was formed, it was deposited on mica in order to analyze by AFM and spectroscopic ellipsometry.

#### 3.2.2. Atomic force microscopy analysis

Analysis of the Langmuir-Blodgett monolayers by atomic force microscopy (AFM) was carried out on an atomic force microscope (Veeco diMultimode V; Veeco, Santa Barbara, CA, USA) operating in air room temperature and equipped with a medium-range scanner (15 × 15 μm^2^ X-Y and 2.3 μm Z). AFM was operated in tapping mode at a scan rate of 1 Hz using RTESP Model tips. NanoScope Analysis v1.2 software by Veeco was used for image analysis and obtain the maximum average area and the maximum average height. Three points of each film surface were scanning to confirm the reproducibility of AFM images.

#### 3.2.3. Spectroscopic Ellipsometry Monolayer-Thickness Measurements

Spectroscopic ellipsometry analysis of the monolayers was determined using an UVISEL LT M200 AGMS Ellipsometer (HORIBA Jobin Yvon; Irvine, CA, USA). After removal of the monolayer from the deposition chamber, the samples were analyzed and the thicknesses of the monolayer determined from a three-phase model using a real refractive index of 1.45 for the monolayer and the previously measured complex refractive indexes for the substrate (mica) (Figure 6). Algorithms for these calculations have been previously described.

#### 3.2.4. Isolation of 10,16-dihydroxypalmitic acid (10,16-DHPA, **1**)

10,16-DHPA was obtained by depolymerization of tomato cutin, obtained from agroresidual wastes, as previously described [15,16], and its structural characterization was determined by comparing the NMR and MS data with those previously reported [16].

#### 3.2.5. Oxidation of 10,16-DHPA (**1**) with Pyridinium Chlorochromate (PCC)

To a stirred solution of PCC (0.23 mmol) in CH_3_CN, 10,16-DHPA (compound 1) (0.12 mmol) dissolved in CH_3_CN was added. After 24 h, the reaction was stopped and filtered on celite. The solvent was evaporated under vacuum, and the residue was redissolved in ethyl ether, washed with brine, dried, and concentrated to give mainly 7-oxohexadecanedioic acid (ω-carboxyl oxo PA) (**2**). Brown solid, (32 mg, 63% yield). ^1^H-NMR (750 MHz, CDCl_3_) δ 2.36 (m, 8H) CH_2_-2,6,8 and CH_2_-15, 1.62 (m, 8H) CH_2_-3,5,9 and CH_2_-14, 1.30 (m, 10H) CH_2_-4,10,11,12 and CH_2_-13. ^13^C-NMR (187.5 MHz, CDCl_3_) δ 211.73 (CO-7), 180.23 (COOH-16), 179.96 (COOH-1), 42.94 (C-8), 42.52 (C-6), 34.16 (C-2), 33.96 (C-15), 29.21, 29.09, 28.90, 28.67, 24.72, 24.53, 24.37, 23.91 and 23.49 (C-3 to C-5 and C-9 to C-14) EI-HRMS *m*/*z* 300.1944 (C_16_H_28_O_5_, 300.1937). EI-MS *m*/*z* 300 [M]^+^, 241 [M−C_2_H_3_O_2_]^+^, 185 [M−C_6_H_11_O_2_]^+^, 157 [M−C_7_H_11_O_3_]^+^ [16].

#### 3.2.6. Polyesterification of ω-Carboxyl-oxo-PA and 1,8-Octanediol

Reactions were performed in a shaking incubator (VorTemp^TM^ 1550, Labnet International, Edison, NJ, USA) in toluene at 60 °C. ω-carboxyl-oxo PA (2, 20 mg, 665 mM), 1,8-octanediol (3, 9.7 mg, 665 mM) and lipase (10%-by-wt relative to monomer) (Scheme 1) were added to capped reaction vials (5 mL). The vials were shaken at 120 rpm for 24 and 48 h at 60, 70 and 80 °C. Reactions without the addition of lipase were used as controls. In a different series of experiments, activated molecular sieves (20 mg Merck (Toluca, Mexico) 4 Å) were added to the vials to remove water. Once the reaction was completed, it was stopped by adding an excess of cold chloroform, then the reaction mixture was filtered to remove the enzyme and the solvent was concentrated under reduced vacuum. Polyesters were extracted with chloroform and were characterized by NMR, MS and AFM analysis.

#### 3.2.7. Copolyester from ω-carboxyl-oxo-PA and 1,8-octanediol

(Poly(*ω*-carboxyl PA-*co*-OD)). Yield: 80% (4), ^1^H-NMR (750 MHz CDCl_3_) δ ppm 4.05 (t, 2H) -O-CH2-, 3.63 (m, 4H) C(OH)H-b, C(OH)H-b′, C(OH)H-c and C(OH)H-c′, 2.27 (t, *J* = 7.52 Hz, 2H) CH_2_-a and CH_2_-a′, 1.60–1.22 (m) –CH_2_-. ^13^C-NMR (125 MHz, CDCl_3_) δ 177.68 (CO); 72.40 (C-b and C-b′); 63.00 (C-c and C-c′).

#### 3.2.8. Conversion of ω-Carboxyl-oxo-PA and 1,8-Octanediol

In order to know the conversion of ω-carboxyl-oxo-PA and 1,8-octanediol by CAL-B versus time, ω-carboxyl-oxo-PA (2, 20 mg, 665 mM), 1,8-octanediol (3, 9.7 mg, 665 mM) and lipase (10%-by-wt relative to monomer) (Scheme 1) were added to capped reaction vials (5 mL). Toluene (1 mL) was used as a solvent and the sample was placed in a shaking incubator (VorTemp™ 1550; Labnet International, Edison, NJ, USA). The vials were shaken at 120 rpm at 60 °C and samples of the reaction were taken at 0, 1, 4, 8, 12, 24 and 48 h to be analyzed with electrospray in positive mode (ESI+) by direct injection. The reactions were stopped by adding an excess of cold chloroform and then removing the enzyme by filtration (glass-fritted filter, medium porosity). These non-fractionated products (no precipitated) were analyzed by MALDI-TOF and characterized by ^1^H-NMR to determine their molecular-weight distribution and to analyze the different species generated.

## 4. Conclusions

In this study, a polyester (poly(*ω*-carboxyl PA-*co*-OD)) with M_w_ = 2155.15 and M_n_ = 1637.27 was synthesized from ω-carboxyl-oxo-PA and 1,8-octanediol in a good yield. The best enzymatic reaction conditions to get the polyester were using lipase CAL-B in toluene as a solvent for 1 h at 60 ⁰C. The polyester characterization by ^1^H-NMR and MALDI-TOF showed a linear polymer which has a good organization forming a homogeneous film. The obtained film showed a porous surface which can be used as an anchor point to potentially be used as tissue scaffolds. This work contributes to the study of the use of long-chain aliphatics to obtain biopolyesters with potential use in the biomedical field as a flexible platform for tissue engineering or drug delivery strategies.

## Figures and Tables

**Table 1:** M_w_ and yields of the copolymerization reaction at different temperatures.

Reaction Time (h)	60 °C (% Yield)	70 °C (% Yield)	80 °C (% Yield)
24	^1^1922.85 ± 1.03 (98)	1897.67 ± 0.54 (95)	1638.15 ± 0.45 (90)
48	1761.16 ± 0.98 (96)	1592.55 ± 1.10 (90)	1649.86 ± 0.88 (96)
72	1849.87 ± 0.99 (97)	1414.84 ± 0.87 (93)	1455.92 ± 0.78 (95)

^1^ M_w_ average of 3 different reactions analyzed by MALDI-TOF.

**Table 2:** M_w_, M_n_ and PDI (Mw/Mn) Values for copolymerization of ω-carboxyl oxo PA and 1,8-octanediol using Novozyme 435.

Reaction Time (h)	M_w_	M_n_	PDI
1	2155.15 ± 0.45	1637.27 ± 0.75	1.31 ± 0.05
2	1798.99 ± 0.65	1451.17 ± 0.80	1.23 ± 0.09
3	1895.86 ± 0.23	1619.55 ± 0.19	1.17 ± 0.08
4	1749.37 ± 0.45	1468.86 ± 0.37	1.19 ± 0.11
5	1796.22 ± 0.19	1416.08 ± 0.15	1.26 ± 0.10
8	1919.24 ± 0.15	1599.27 ± 0.20	1.20 ± 0.32
10	1598.51 ± 0.34	1369.11 ± 0.25	1.16 ± 0.07
24	1897.67 ± 0.28	1548.37 ± 0.20	1.22 ± 0.06
48	1592.55 ± 0.19	1363.76 ± 0.15	1.16 ± 0.05

^1^ Mw, weight-average molecular weight; Mn, number-average; PDI (M_w_/M_n_): polydispersity index.
